# Open-Source Technology for Real-Time Continuous Glucose Monitoring in the Neonatal Intensive Care Unit: Case Study in a Neonate With Transient Congenital Hyperinsulinism

**DOI:** 10.2196/21770

**Published:** 2020-12-04

**Authors:** Katarina Braune, Mandy Wäldchen, Klemens Raile, Sigrid Hahn, Tebbe Ubben, Susanne Römer, Daniela Hoeber, Nora Johanna Reibel, Michael Launspach, Oliver Blankenstein, Christoph Bührer

**Affiliations:** 1 Charité – Universitätsmedizin Berlin Department of Paediatric Endocrinology and Diabetes Berlin Germany; 2 Berlin Institute of Health Berlin Germany; 3 School of Sociology University College Dublin Belfield Ireland; 4 Charité – Universitätsmedizin Berlin Department of Neonatology Berlin Germany; 5 #dedoc° Diabetes Online Community Dedoc Labs GmbH Berlin Germany; 6 Charité – Universitätsmedizin Berlin Department of Paediatric Gastroenterology, Nephrology and Metabolic Diseases Berlin Germany; 7 Charité – Universitätsmedizin Berlin Institute for Experimental Paediatric Endocrinology Berlin Germany; 8 Charité – Universitätsmedizin Berlin Newborn Screening Laboratory Berlin Germany

**Keywords:** open-source, mobile health, continuous glucose monitoring, off-label use, neonatal hypoglycemia, congenital hyperinsulinism, transient hyperinsulinism

## Abstract

**Background:**

Use of real-time continuous glucose monitoring (rtCGM) systems has been shown to be a low-pain, safe, and effective method of preventing hypoglycemia and hyperglycemia in people with diabetes of various age groups. Evidence on rtCGM use in infants and in patients with conditions other than diabetes remains limited.

**Objective:**

This case study describes the off-label use of rtCGM and the use of an open-source app for glucose monitoring in a newborn with prolonged hypoglycemia secondary to transient congenital hyperinsulinism during the perinatal period.

**Methods:**

The Dexcom G6 rtCGM system (Dexcom, Inc) was introduced at 39 hours of age. Capillary blood glucose checks were performed regularly. In order to benefit from customizable alert settings and detect hypoglycemic episodes, the open-source rtCGM app xDrip+ was introduced at 9 days of age.

**Results:**

Time in range (45-180 mg/dL) for interstitial glucose remained consistently above 90%, whereas time in hypoglycemia (<45 mg/dL) decreased. Mean glucose was maintained above 70 mg/dL at 72 hours of life and thereafter. Daily sensor glucose profiles showed cyclic fluctuations that were less pronounced over time.

**Conclusions:**

While off-label use of medication is both common practice and a necessity in newborn infants, there are few examples of off-label uses of medical devices, rtCGM being a notable exception. Real-time information allowed us to better understand glycemic patterns and to improve the quality of glycemic control accordingly. Severe hypoglycemia was prevented, and measurement of serum levels of insulin and further lab diagnostics were performed much faster, while the patient’s individual burden caused by invasive procedures was reduced. Greater customizability of threshold and alert settings would be beneficial for user groups with glycemic instability other than people with diabetes, and for hospitalized newborn infants in particular. Further research in the field of personal and off-label rtCGM use, efficacy studies evaluating the accuracy of low glucose readings, and studies on the differences between algorithms in translating raw sensor data, as well as customization of commercially available rtCGM systems, is needed.

## Introduction

Transient and persistent congenital hyperinsulinism (CHI) are rare diseases affecting 1 to 2 in 50,000 newborns. At least 12 genetic defects have been identified that result in dysregulated and increased insulin secretion and subsequently severe hypoglycemia, which may be transient or persistent [1,2]. Frequent hypoglycemia is a potentially life-threatening complication of CHI and may lead to permanent brain damage, which may present in developmental delay and mild to severe neurocognitive difficulties such as deficits in attention, memory, and visual and sensorimotor functions in children with CHI [3,4]. Furthermore, prolonged hospitalizations and intense medical regimes pose a constant psychological burden to infants and children with CHI and their caregivers, limiting their participation in social life, kindergarten, and school [2,5]. Therefore, prevention of hypoglycemia is the main treatment goal in patients with CHI [5,6]. Therapeutic regimens are complex and depend on the underlying genotype and phenotype. Treatment options today include regular feeding, pharmacotherapy with diazoxide, somatostatin analogues and glucagon for diffuse forms, and surgery for focal variants of CHI [6].

Real-time continuous glucose monitoring (rtCGM) and intermittently scanned continuous glucose monitoring (isCGM), as designed for and commonly used by people with diabetes, provide information on current interstitial glucose levels and trends, and alert the user of current or predicted hypoglycemia and hyperglycemia as well as rapid changes in glucose levels. With regular measurements up to every 5 minutes, they also provide statistics on time spent in hypoglycemia, in hyperglycemia, and within range.

Evidence on rtCGM use for glycemic management in neonates is limited, and there is no evidence of calibration-free rtCGM systems in infants so far. Benefits with respect to glycemic control [7] and less procedural pain [8] have been previously described for very low-birth-weight preterm infants in a feasibility study. Furthermore, a study found continuous tissue glucose monitoring to be helpful to identify infants at risk for metabolic instabilities [9]. However, knowledge remains limited for infants and other patient groups at risk of hypoglycemia so far [10,11].

The off-label use of rtCGM in children with CHI was first described in 2004 [12]. Further studies describe rtCGM and isCGM as safe, effective, convenient, and less painful methods of glucose monitoring in CHI patients that provide helpful information for therapeutic decisions [13,14]. Caregivers found the indication of glucose trends to be helpful, felt more confident in everyday management of the condition, and felt less worried about the occurrence of asymptomatic hypoglycemia [12,14]. However, older rtCGM/isCGM systems were reported to have limited accuracy, especially for hypoglycemic levels, and therefore not recommended to completely replace capillary blood glucose (CBG) testing, but rather seen as an adjunctive, beneficial therapy [13,15].

To our knowledge, this case study describes the first use of the rtCGM app xDrip+ in a hospital and intensive care unit (ICU) setting and the first off-label use of rtCGM in a newborn with severe hypoglycemia secondary to transient CHI soon after birth.

## Methods

### Clinical Case

A male infant was delivered full term with a birth weight of 3575 g. An emergency cesarean delivery was performed in response to fetal heart rate monitoring indicating fetal distress. The infant presented with bradypnea, floppiness, and sporadic myoclonic jerks and was transiently treated with noninvasive positive pressure resuscitation. A CBG level of 12 mg/dL was detected at 60 minutes of life. Despite feeding attempts and buccal administration of 40% dextrose gel, the blood glucose level decreased further to 1 mg/dL at 120 minutes of life. The patient was started on continuous intravenous glucose and admitted to the neonatal intensive care unit (NICU) for further diagnostics and treatment. The continuous intravenous glucose administration required to maintain blood glucose levels above 50 mg/dL was incrementally increased from 8 mg/kg/min to over 18 mg/kg/min. This required placement of a central venous line at 37 hours of age to allow for administration of hypertonic glucose solutions. In addition, the infant received copious enteral feedings.

The child’s father and the paternal uncle were both reported to have experienced transient postnatal hypoglycemia, requiring hospitalization for 3 weeks after birth. The child’s father reported having often experienced mild hypoglycemia symptoms such as hunger and craving for sweets when fasting. However, blood glucose levels were reported to be within normal range during routine checkups as an adult. There was no history of diabetes in either of the parents, and screening for gestational diabetes during pregnancy was reported to be negative. However, the paternal grandmother was reported to have been diagnosed with type 1 diabetes at 7 years of age.

### Genetic Testing

Next-generation sequencing (quality level type A; SureSelect XT, Custom Constitutional Panel 17 Mb; Agilent Technologies, Inc) of a CHI panel, including *ABCC8* (GenBank NM_000352.4), *HADH* (GenBank NM_005327.4), *HNF4A* (GenBank NM_175914.4), *KCNJ11* (GenBank NM_000525.3), *KMT2D* (GenBank NM_003482), and *UCP2* (GenBank NM_003355.2), was performed by Labor Berlin, Germany.

### Real-Time Glucose Monitoring

**Figure 1:**
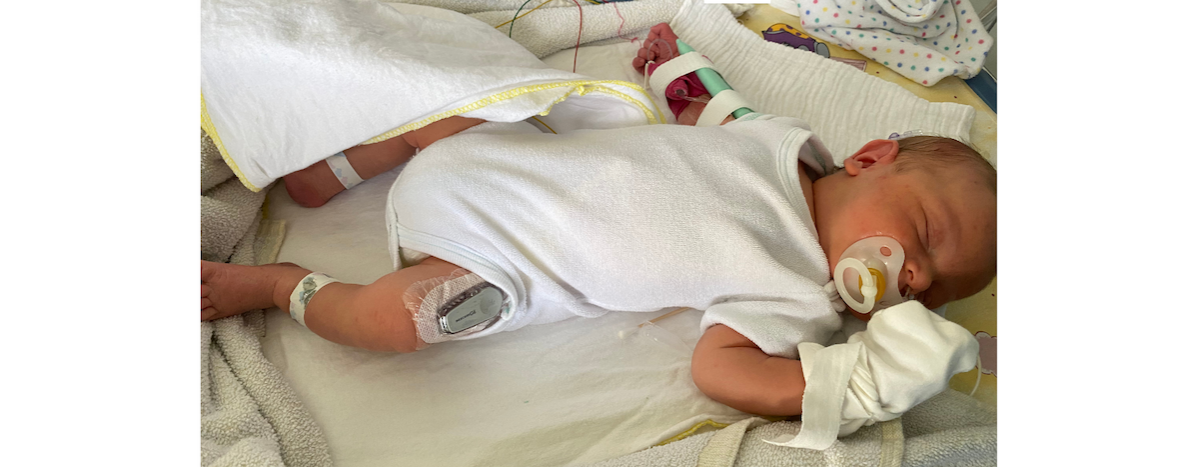
Upper thigh as a suitable rtCGM application site in neonates.

**Figure 2:**
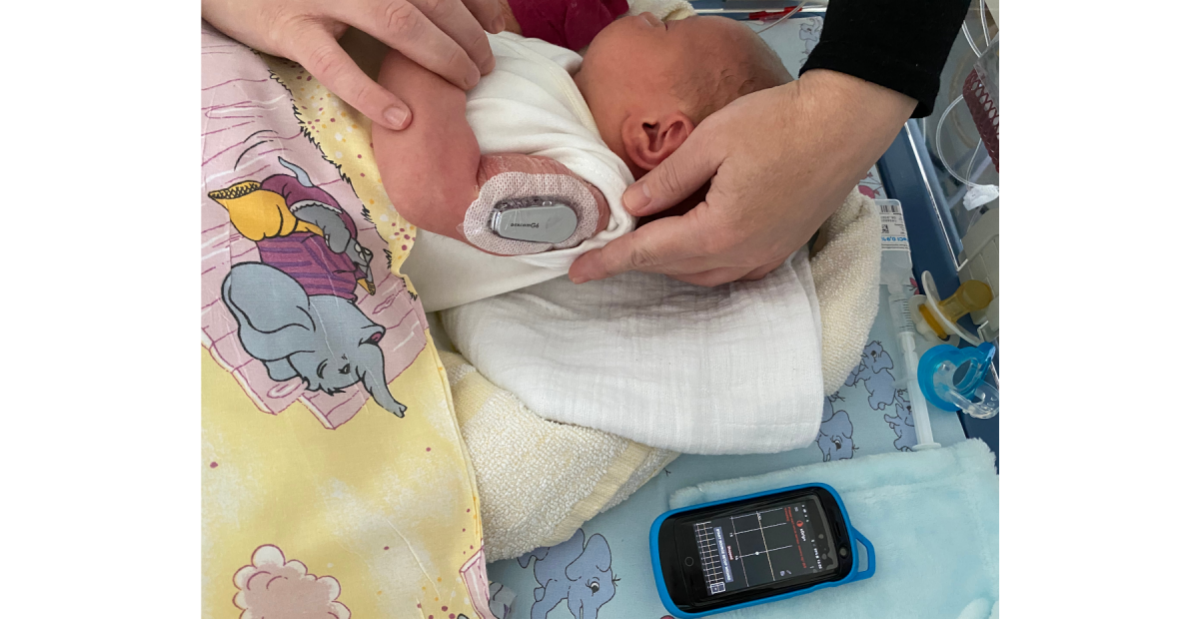
Upper arm as a suitable rtCGM application site in neonates.

To reduce the frequency of capillary blood samples and estimate the right moment for further lab diagnostics, the Dexcom G6 rtCGM system (Dexcom, Inc) was introduced for off-label use at 39 hours of age. In addition, CBG checks were performed regularly, at least 3 times per day (Multimedia Appendix 1). All decisions concerning the therapeutic regimen were based on confirmatory CBG tests. Sensors were placed at the lateral sides of thighs or upper arms (Figures 1 and 2) where enough fat and muscle tissue were present. Sensors were replaced every 7 days.

During the first week, the sensor was paired with a Dexcom G6 handheld device with customizable alert settings; however, the urgent low glucose alarm threshold at a glucose level of 55 mg/dL, which was frequently passed in this neonate but does not meet the requirements in neonates within the first week of life, could not be further reduced. Furthermore, glucose levels below 40 mg/dL could not be further differentiated. In order to benefit from customizable alert settings and include potential hypoglycemic levels below 40 mg/dL, the informed decision was made to pair the sensors with the caregivers’ personal Android phone using the app xDrip+.

### Specifics of the xDrip+ Algorithm

xDrip+ [16] is an open-source product with no regulatory approval that allows the user to choose between the approved native algorithm, which runs on the Dexcom G6 transmitter for translating uncalibrated transmitter data into interstitial glucose level estimations, and its own algorithm, which approximates a linear function using the method of weighted least squares regression by weighting calibration points based on their value, sensor and calibration age, the current glucose trend, a variability heuristic, and other factors. Furthermore, low glucose alarm settings are fully customizable. In this case study, the xDrip+ algorithm was used with a minor modification that allowed sensor glucose levels below 40 mg/dL to be received, although accuracy was expected to be limited in this range. Alerts were set at 45 mg/dL for low sensor glucose levels and at 140 mg/dL for high sensor glucose levels.

### Data Analysis

For the first 7 days of sensor data, rtCGM readings were exported from the Dexcom handheld device using the Dexcom Clarity software. For the following days when xDrip+ was used, sensor data was exported through the built-in data export function in the xDrip+ app. For the analysis of sensor data, we computed statistical properties (arithmetic mean and standard deviation) in the programming language Python 3.7. Furthermore, time in hypoglycemia and time in range were calculated as follows:

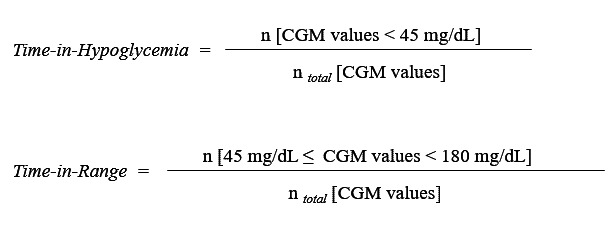



As there is currently no standardized definition for treatment consequences in response to low and high glucose levels in neonates, but their physiological range tends to be lower than in older individuals [9], sensor glucose in range was defined by a lower threshold of 45 mg/dL (2.5 mmol/L) and an upper threshold of 180 mg/dL (10 mmol/L). Therefore, time in hypoglycemia was calculated based on sensor readings under 45 mg/dL (2.5 mmol/L).

## Results

### Diagnostic Workup

The infant responded well to a single subcutaneous injection of 30 µg/kg of glucagon (GlucaGen Hypokit; Novo Nordisk A/S). Therefore, a glycogen storage disease was unlikely to be the underlying condition and congenital hyperinsulinism was suspected. According to CHI treatment guidelines [6], a continuous subcutaneous glucagon infusion was initiated at 64 hours of age and gradually increased (up to 27.2 µg/kg/h) (Multimedia Appendix 1). Glucagon dosage was adjusted (increased or lowered) according to rtCGM glucose using defined thresholds for each action; thus, the continuous glucose infusion could be gradually reduced and completely terminated by 13 days of age. In the following days, glucagon administration was gradually decreased and completely terminated 17 days of age. That treatment algorithm successfully prevented occurrence of any symptomatic hypoglycemia (eg, seizures, hypothermia, hypotonia, bradypnea). No other drugs such as diazoxide or lanreotide were administered.

Initial hypoglycemia screening revealed hyperinsulinemia with no ketonemia present (blood glucose 29 mg/dL, insulin 9.03 mU/L, C-peptide 2.34 µg/L). After terminating the intravenous glucose and the subcutaneous glucagon infusion and with increasing improvement of oral feeding and hence regular and reliable enteral glucose uptake, no new hypoglycemic episodes occurred between days 17 and 20. Consequently, we suspected normalization of insulin secretion, which was substantiated by an analysis after a strict fasting period of 6.5 hours (blood glucose 86 mg/dL, insulin 1.5 mU/L, C peptide 0.54 µg/L, ketone bodies 0.4 mmol/L) on day 20. With these results, we were able to terminate rtCGM use and discharge the infant on the same day in good health.

Genetic testing did not reveal any disease-relevant pathologic variation in genes that are most frequently associated with CHI: *ABCC8* (GenBank NM_000352.4), *HADH* (GenBank NM_005327.4), HNF4A (GenBank NM_175914.4), *KCNJ11* (GenBank NM_000525.3), and *UCP2* (NM_003355.2). However, we detected a novel, highly conserved, and heterozygous missense variant in the *KMT2D* gene (c.3976C>T, R1326W), which was interpreted as a pathogenic variant within this analysis and might be causative in this particular case of a Kabuki syndrome with predominantly transient, congenital hyperinsulinism [17,18].

### Glycemic Outcomes

Time in range (sensor glucose readings between 45 and 180 mg/dL or 2.5 and 10 mmol/L) remained consistent above 90%, whereas time in hypoglycemia (<45 mg/dL or <2.5 mmol/L) decreased, and mean glucose was maintained above 70 mg/dL or 3.8 mmol/L at 72 hours of life and after (Table 1). Daily sensor glucose profiles (Multimedia Appendix 1) show cyclic fluctuations exceeding 45 mg/dL (2.5 mmol/L) on most days and occasionally 180 mg/dL (10 mmol/L). These cyclic fluctuations were found to be less pronounced over time.

rtCGM readings were found to be mostly accurate compared to CBG (Multimedia Appendix 1). Significant deviations (>15%) occurred within the first 24 hours after a new sensor was placed. After recalibration of the rtCGM, accuracy of rtCGM readings was satisfactory (<15% deviations).

**Table 1:** Analysis of rtCGM readings within the first 16 days of life in a neonate with transient CHI, including mean sensor glucose (SD), time in hypoglycemia (defined as the percentage of sensor readings below 45 mg/dL), and time in range (defined as the percentage of sensor readings between 45 mg/dL and 180 mg/dL).

Patient age, d (h)	Sensor readings, n	Glucose, mg/dL, mean (SD)	Readings in range, n	TIR^a^, %	Readings in hypoglycemia, n	TIH^b^, %
2 (39-48)	74	68 (16)	71	95.9	3	4.1
3 (49-72)	291	69 (29)	240	82.5	51	17.5
4 (73-96)	288	98 (34)	288	100.0	0	0.0
5 (97-120)	290	79 (16)	286	98.6	4	1.4
6 (121-144)	291	82 (17)	280	96.2	11	3.8
7 (145-168)	282	94 (36)	274	97.2	8	2.8
8 (169-192)	287	77 (21)	270	94.1	17	5.9
9 (193-216)	203	85 (29)	190	93.6	13	6.4
10 (217-240)	213	81 (22)	197	92.5	16	7.5
11 (241-264)	284	81 (25)	261	91.9	23	8.1
12 (265-288)	281	86 (24)	274	97.5	7	2.5
13 (289-312)	296	81 (19)	284	95.9	12	4.1
14 (313-336)	288	73 (20)	265	92.0	23	8.0
15 (337-360)	248	72 (18)	235	94.8	13	5.2
16 (361-372)	138	83 (17)	138	100.0	0	0.0

^a^TIR: time in range.

^b^TIH: time in hypoglycemia.

## Discussion

### Principal Findings

This case study describes the use of a Dexcom G6 rtCGM system in a neonate with transient CHI during the perinatal period, using two different algorithms. The real-time information provided by the rtCGM system allowed us to better understand glycemic patterns of the underlying condition and to improve the quality of glycemic control of our patient accordingly. Severe hypoglycemia was successfully prevented and measurement of serum levels of insulin and further lab diagnostics were performed much faster, while the patient’s individual burden caused by invasive procedures could be reduced.

To our knowledge, this is the first report on the use of open-source apps for glucose monitoring such as xDrip+ in a hospital and ICU setting, as well as the first study reporting an off-label use of a precalibrated rtCGM system in a neonate. Furthermore, we described the first use of rtCGM in an infant with transient CHI in the most critical phase during the first days of life.

While off-label use of medication is both common practice and a necessity in newborn infants, there are few examples of off-label uses of medical devices, rtCGM being a notable exception [7,8,19-24]. CBG tests are the current standard of care in the management of neonatal hypoglycemia. However, CBG has notable limitations. Heel pricks, associated with pain, are required every time a blood sample is taken. Furthermore, it provides only single point measurements without a continuous profile on glucose trends, dynamics, and interactions with drugs and meals. Conversely, rtCGM records any hypoglycemic episode in a quantitative fashion, displays detailed pharmacodynamics of single drugs, and consequently can help to determine a drug’s long-term requirement. We assume that rtCGM use could be extremely helpful in any hospital or home setting and in this case, could help reduce the child’s risk for neurological long-term complications [3,4,25]. Our experiences with the presented case indicate that greater customizability of threshold and alert settings would be beneficial for user groups with glycemic instability other than people with diabetes, and for hospitalized infants in particular. Further potential use cases may include children of mothers with gestational diabetes, children with metabolic disorders, children with neonatal diabetes mellitus, preterm infants, and infants with difficulties in enteral feeding.

### Strengths and Limitations

It is important to note that although rtCGM provides information about glucose dynamics and variability, these systems might be less accurate when it comes to direct comparison with point-of-care testing of blood glucose levels. In order to keep invasive diagnostics for the patient reduced to a minimum, there were not enough CBG data points available to allow for a robust calculation of the mean absolute relative difference. This limits our ability to evaluate the accuracy of rtCGM readings, particularly of glucose levels <40 mg/dL. As rtCGM systems correlate the electric signal of the sensor with interstitial glucose concentrations in a linear fashion, they are not designed to detect the low glucose levels that occur in newborn infants after birth. Moreover, alert settings are not fully customizable to threshold values that seem reasonable to use in infants, who have a much lower physiological glucose range compared to older children and adults [9].

The app xDrip+, which was used in this case study, originates from an online community of people living with type 1 diabetes. The Android application package needed for setup, the app’s source code, and instructions on how to install and use the app are openly available on the internet. Recently, open-source rtCGM apps such as xDrip+ for Android phones [16] and Spike for Apple iOS [26] have become increasingly popular and are used by thousands of people in the worldwide type 1 diabetes community [27], especially by those living in countries where commercial rtCGM systems are not accessible or not reimbursed by the healthcare system [28]. Despite their popularity, so far neither of the aforementioned open-source rtCGM apps have been tested in clinical trials or been approved by a regulatory body. The creators of these apps and the online community around them publicly disclose that they should be used with caution at all times and at one’s own risk. The controlled environment of a NICU was an ideal setting to observe outcomes and potential benefits of the off-label use while ensuring minimal risk for the patient at the same time. We found that the app was also a more suitable solution that best met the child’s medical needs as well as healthcare professionals’ and the caregivers’ expectations on relevant alarms in case of glucose excursions. However, the safety and accuracy of xDrip+ as such has not been systematically investigated so far, and its use has only been described in conjunction with observational and self-reported data from open-source automated insulin delivery system users [29,30]. Further research on personal and off-label rtCGM use and differences between native and alternative algorithms in translating raw sensor data is needed, as well as customization of commercially available rtCGM systems.

### Conclusions

The use of rtCGM can be considered in neonatal patients at risk of hypoglycemia, such as infants with transient or permanent CHI, to reduce the frequency of blood glucose measurements and focus them on potential decision-making points. However, these devices have been designed to be used by people with diabetes and are currently not approved for use in children younger than 2 years of age. Other use cases, such as infants with glycemic instability and other patients with rare conditions that might benefit from continuous glucose monitoring, are currently not being addressed sufficiently by the medical device market. Further investigation on the use and accuracy of rtCGM in wider patient groups, as well as further customization of rtCGM systems, is needed to address unmet needs of wider population groups.

## Appendix

Multimedia Appendix 1 Daily profiles of sensor glucose and therapeutic management of a newborn with transient CHI. Sensor glucose readings [mg/dL] are shown in blue, i.v. glucose distribution in green [mg/kg/min) and s.c. glucagon infusion in orange [µg/kg/h]. Low sensor glucose threshold (45 mg/dL) is shown in red. Capillary blood glucose (CBG) measurements are marked in magenta.

## Abbreviations

CBG: capillary blood glucose
CHI: congenital hyperinsulinism
ICU: intensive care unit
isCGM: intermittently scanned continuous glucose monitoring
NICU: neonatal intensive care unit
rtCGM: real-time continuous glucose monitoring
