# Supplementary material for: Open-Source Technology for Real-Time Continuous Glucose Monitoring in the Neonatal Intensive Care Unit: Case Study in a Neonate With Transient Congenital Hyperinsulinism
Source: J Med Internet Res. 2020 Dec 4;22(12):e21770. doi: 10.2196/21770 (PMC7748959; doi:10.2196/21770)
Supplement: Multimedia Appendix 1 [file jmir_v22i12e21770_app1.docx]

**Appendix 1:** Daily profiles of sensor glucose and therapeutic management of a newborn with transient CHI. Sensor glucose readings [mg/dL] are shown in blue, i.v. glucose distribution in green [mg/kg/min) and s.c. glucagon infusion in orange [µg/kg/h]. Low sensor glucose threshold (45 mg/dL) is shown in red. Capillary blood glucose (CBG) measurements are marked in magenta.

**A**

**
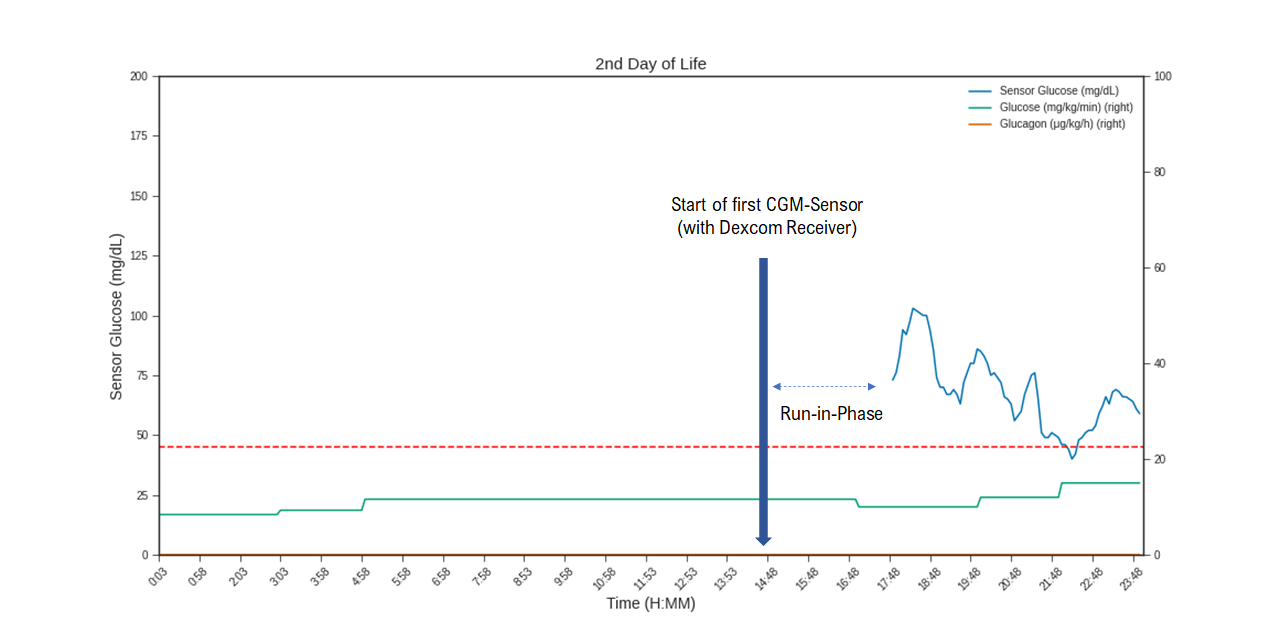
**

**B**

**
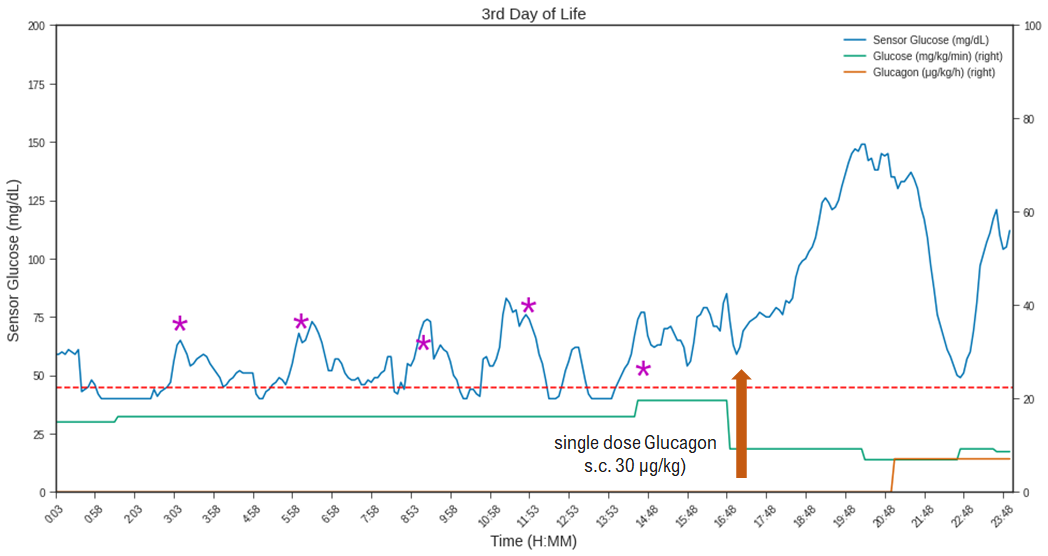
**

**C**

**
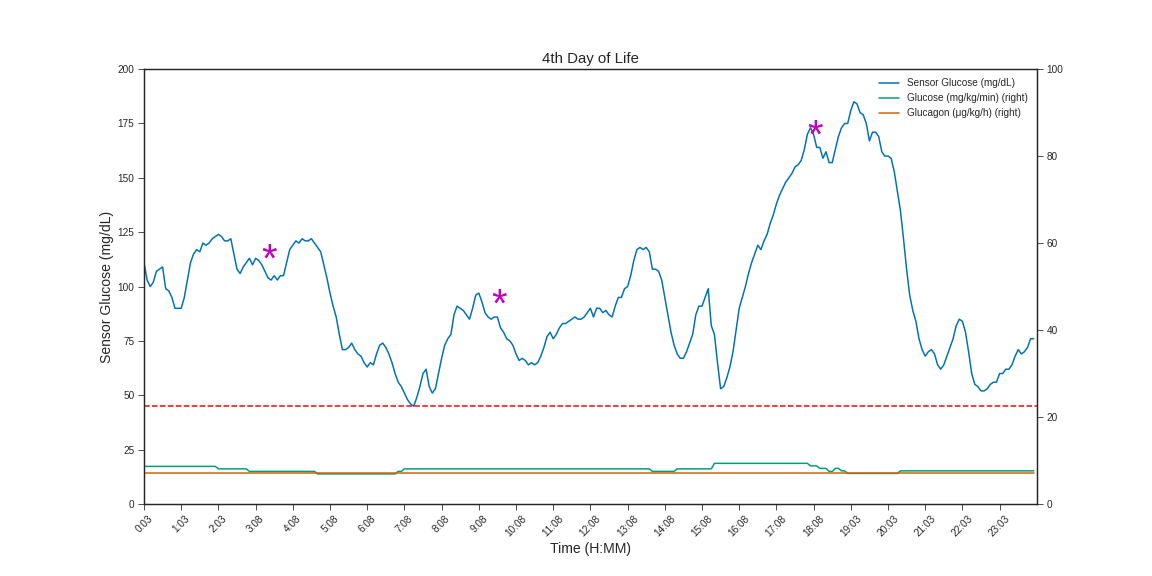
**

**D**

**
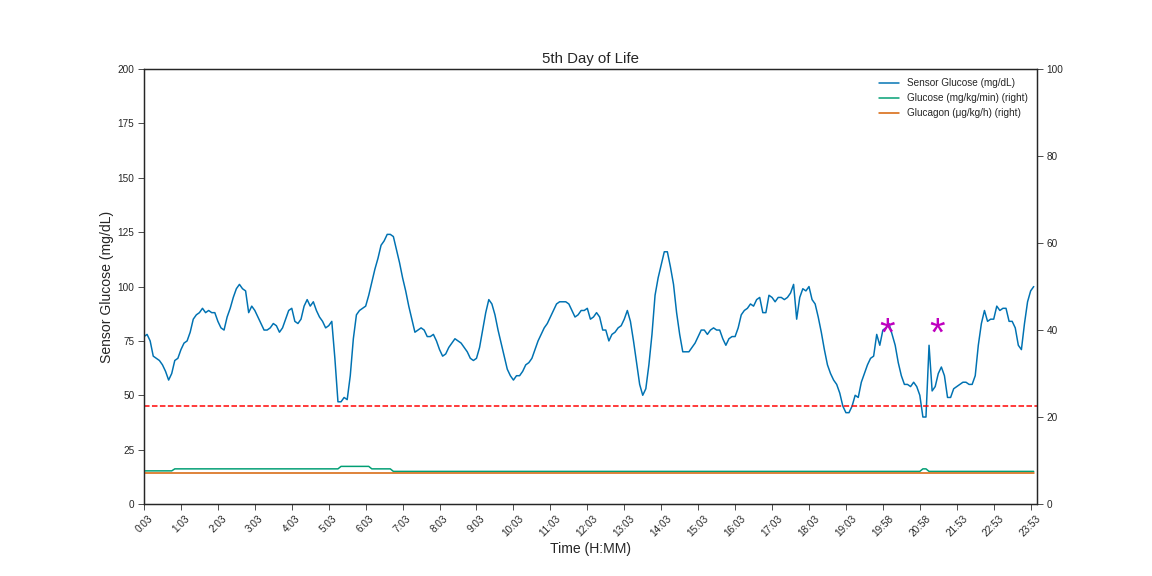
**

**E
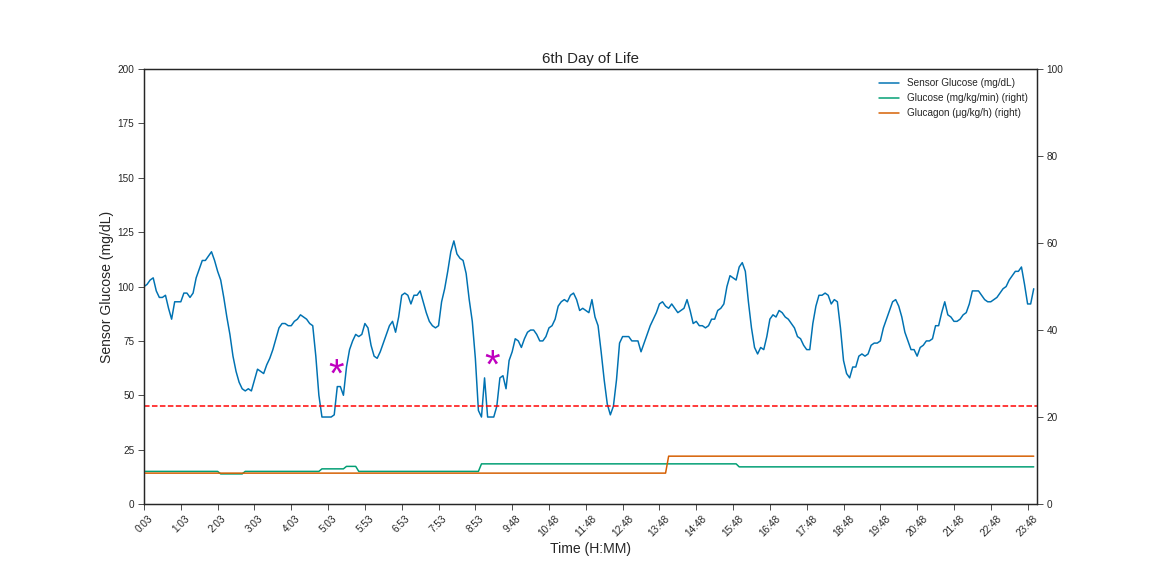
**

**F
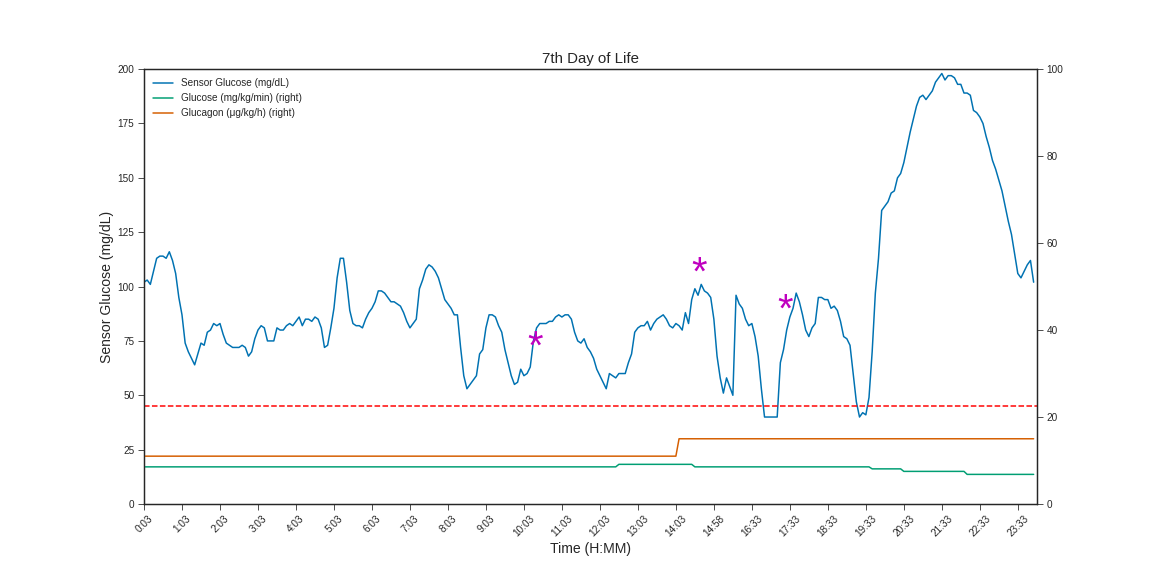
**

**G
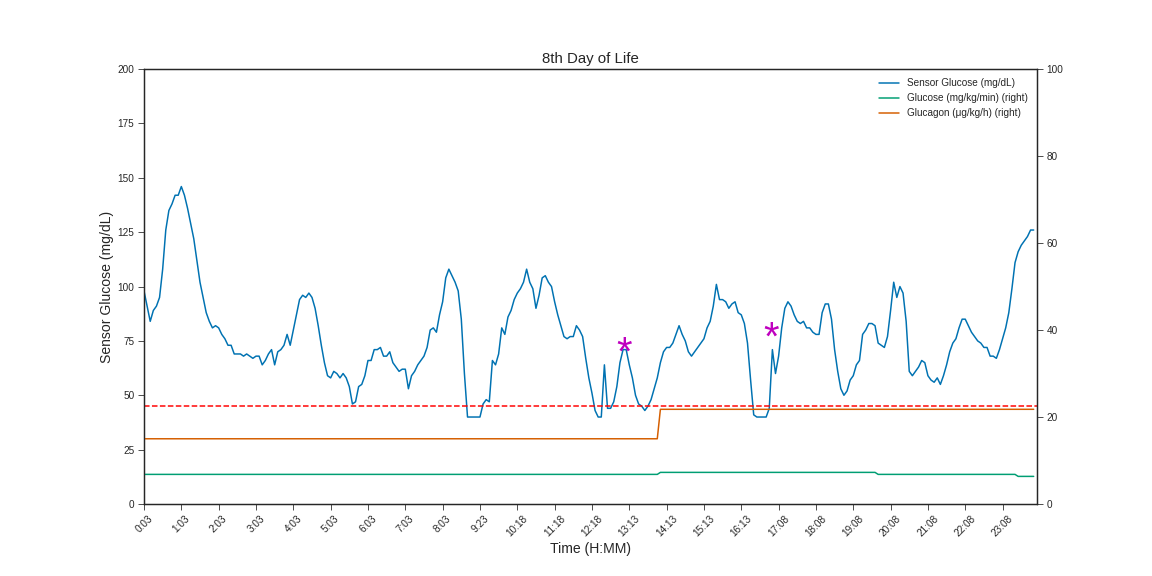
**

**H
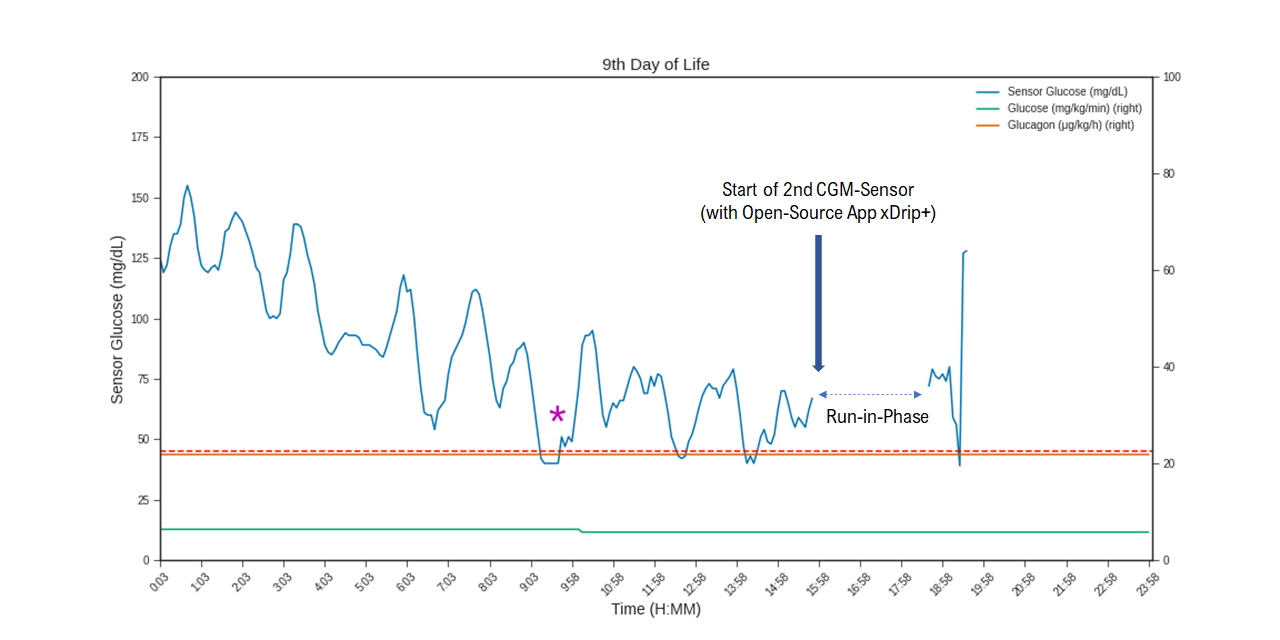
**

**I**

**
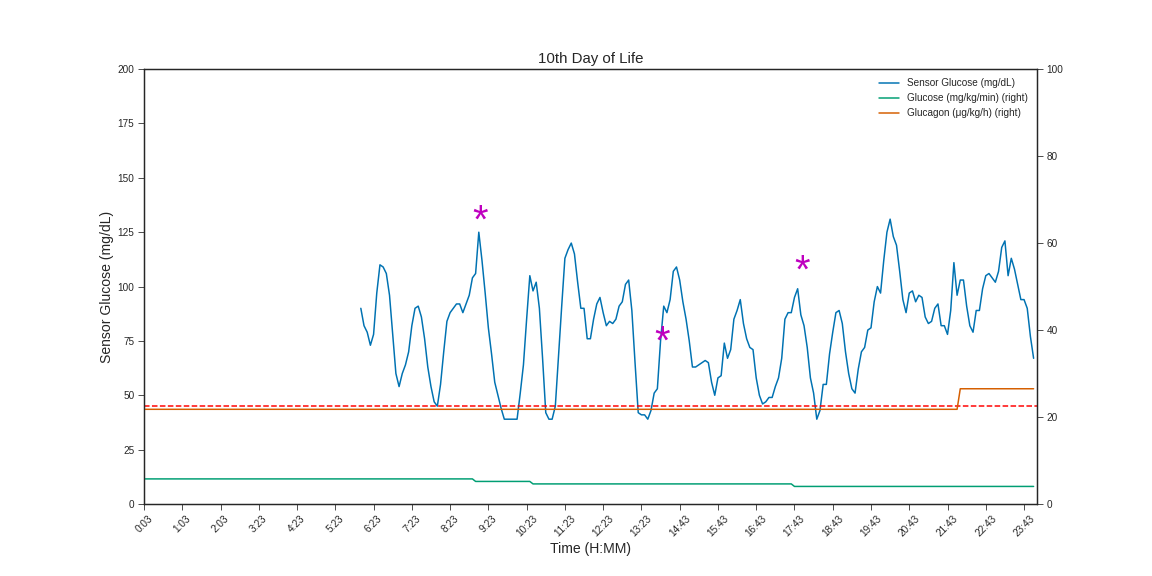
**

**J

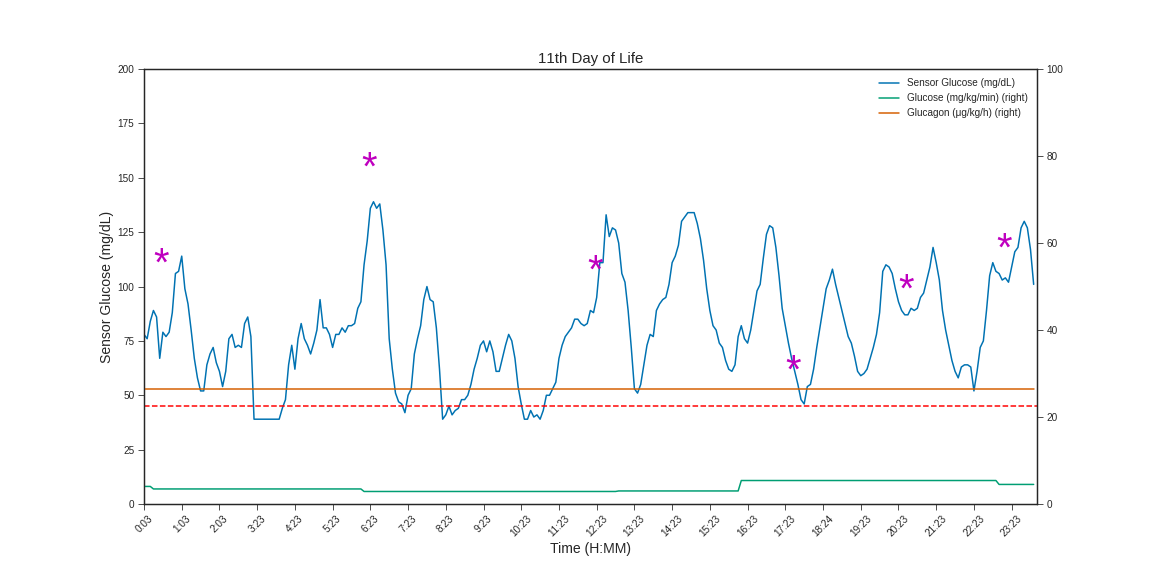
**

**K**

**
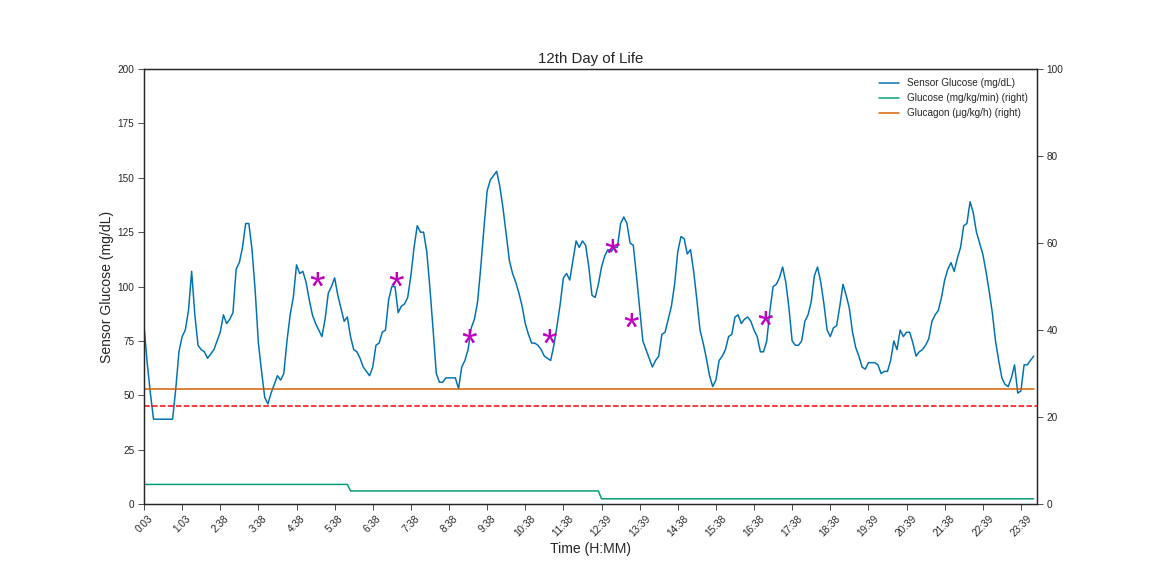
**

**L**

**
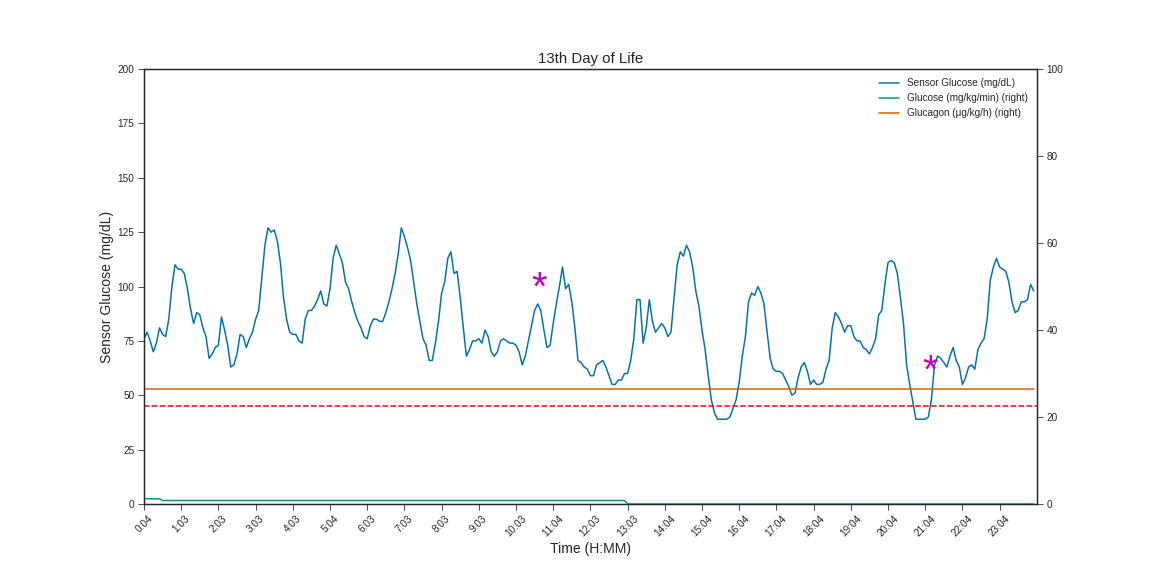
**

**M**

**
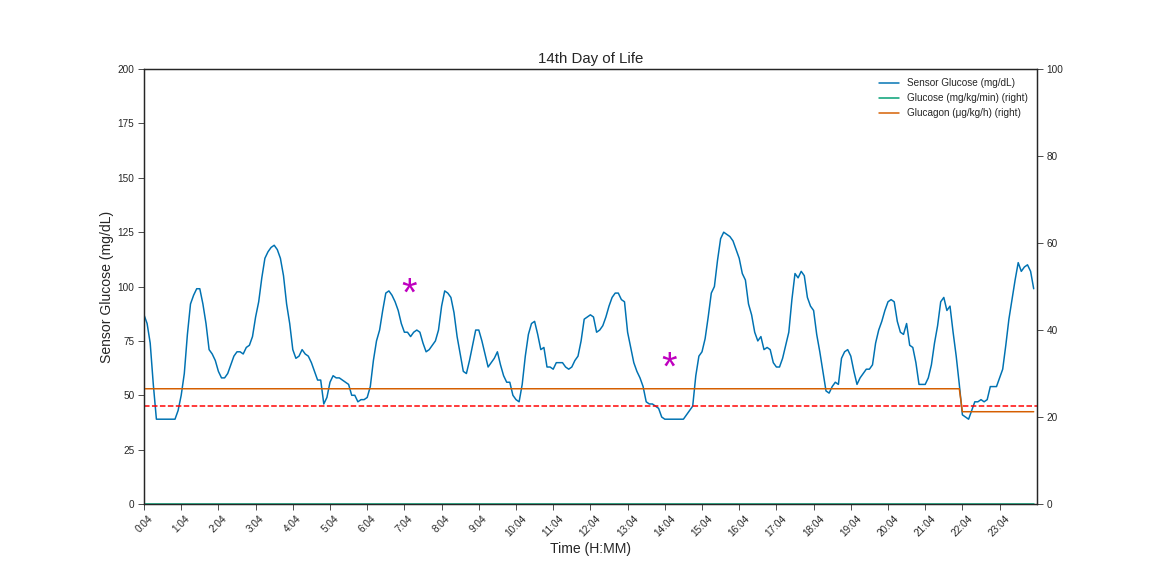
**

**N**

**
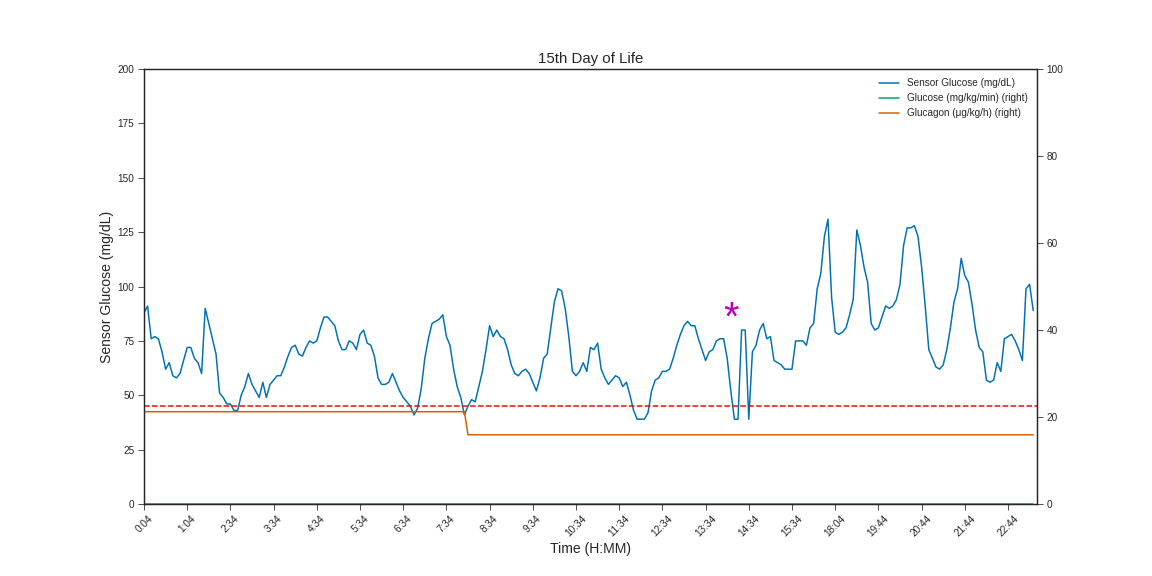
**

**O
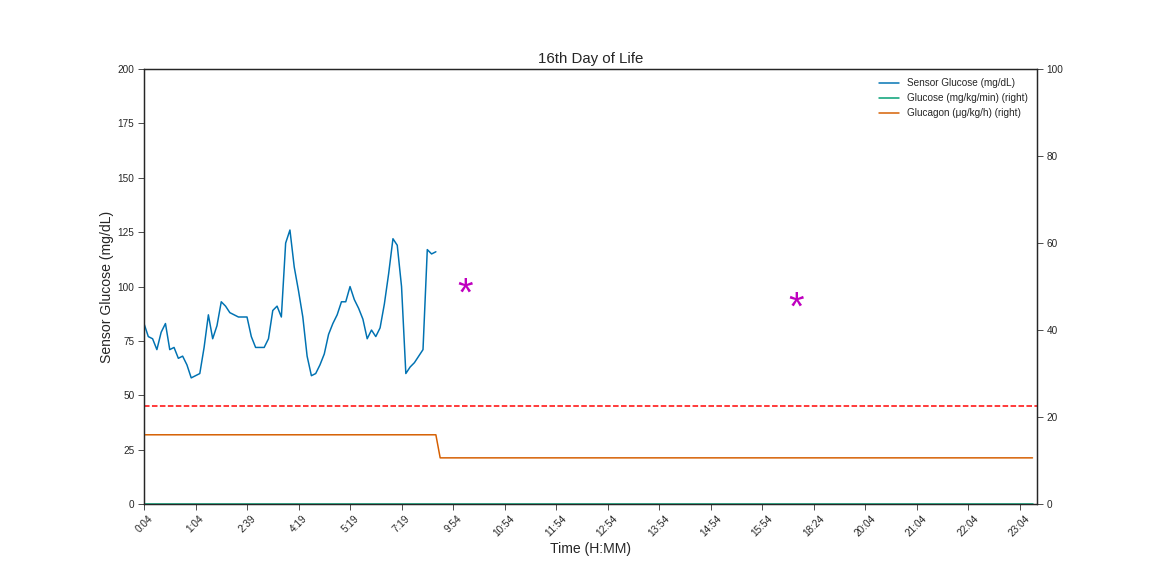
**
